# Involvement of Bcl-2 Activation and G1 Cell Cycle Arrest in Colon Cancer Cells Induced by Titanium Dioxide Nanoparticles Synthesized by Microwave-Assisted Hybrid Approach

**DOI:** 10.3389/fbioe.2020.00606

**Published:** 2020-07-15

**Authors:** Shivendu Ranjan, Nandita Dasgupta, Debasish Mishra, Chidambaram Ramalingam

**Affiliations:** ^1^Faculty of Engineering and the Built Environment, University of Johannesburg, Johannesburg, South Africa; ^2^Bio-Inspired Design Laboratory, School of Bio Sciences and Technology, VIT University, Vellore, India; ^3^Industrial Biotechnology Division, School of Bio Sciences and Technology, VIT University, Vellore, India

**Keywords:** titanium dioxide nanoparticle, cell cycle arrest, Western blot, toxicity, mechanism of action

## Abstract

The toxic effect of TiO_2_ nanoparticles (TNP) greatly varies with the variation in synthesis protocol followed. Any morphological alteration of TNPs affects their activity. In the present study, we report the detailed toxicological analysis of TNPs fabricated by a microwave irradiation–assisted hybrid chemical approach. The toxicological mechanism was studied in human colon cancer cell lines (HCT116). Results indicate that TNP induces oxidative stress on HCT116, which, in turn, causes mitochondrial membrane depolarization. We also observed activation of Bcl-2 and caspase-3 by Western blot analysis. This indicates TNPs induce mitochondrial-mediated apoptosis. Furthermore, G1 cell cycle arrest was observed by flow-cytometric analysis. This study provides an understanding of the mechanism of action for apoptosis induced by TNPs, which can be further used to design safe TNPs for various consumer products and also suggests that extensive research needs to be done on harmful effects of TNPs synthesized from different approaches before commercial application.

## Introduction

Recently, nanotechnology has been explored in various interdisciplinary areas spanning the fields of chemistry, biology, electronics, and medicine for various potential applications. A lot of consumer products have been developed with the use of nanomaterials. Researchers are developing new applications or products with nanomaterials because of their unique properties, such as quantum effect and optical behavior that differentiates them from their bulk counterparts (Qian et al., 2014; Jeevanandam et al., 2018; Sudha et al., 2018). Because the use of nanomaterials has increased considerably and has penetrated into commercial products, there is a need to assess the safety aspect of these nanomaterials. Being smaller than cellular organelles, nanoparticles can easily penetrate cells and interact with biomolecules (Ahamed et al., 2016; Jain et al., 2016; Banks et al., 2018; Dasgupta et al., 2018).

Among nanomaterials, TNPs are employed in a wide range of consumer products. They are used as whitening agents in confectionary products. Bulk TiO_2_ is considered as inert and safe to use. It has been assigned E-number 171 by the EU and is accepted for consumption in food materials in bulk form. However, TNPs have been reported to elicit toxic responses and interact with proteins due to their high reactivity. TNPs, just as other nanoparticles, have been reported to have cytotoxicity, genotoxicity, inflammatory responses, and DNA-damaging effects. The exact mechanism of action is still unknown. A possible mechanism is induction of oxidative stress, which results in the formation of reactive oxygen species (ROS) (Ranjan et al., 2016b; Fadda et al., 2018; Huerta-García et al., 2018; Gea et al., 2019; Pedata et al., 2019; Santonastaso et al., 2019). As such, any possible adverse effects of ingested TNPs need to be examined to rule out any health risk associated with consumption of TNP-containing products. TNP toxicity also depends upon the fabrication method, and thus, each unique fabricated TNP must be analyzed for its toxicological behavior (Hamilton et al., 2009).

In the present study, we analyzed the cytotoxic effects of TNPs (fabricated by a unique hybrid approach) in HCT-116 (human colon cancer) cells. We tried to deduce the mechanism of action, including their potential to induce oxidative stress, such as ROS, DNA damage and cell cycle arrest. We further attempted to probe the probable role of apoptosis-mediated cell death that can be a result of ROS generation. This is the first-of-its-kind study of the detailed cytotoxicological mechanism of titanium dioxide fabricated by a microwave irradiation–assisted hybrid chemical approach on colon cancer cell lines.

## Materials and Methods

### Chemicals

All reagents used were of analytical grade and were used without further purification. Throughout the procedures, double deionized (DI) water (with a measured resistivity of 18.2 MΩcm^−1^) was used. Cisplatin or cis-diamminedichloridoplatinum (CDDP) was used as a positive control.

### TNP Synthesis and Characterization

TNPs were synthesized by a microwave irradiation–assisted hybrid chemical approach. A detailed protocol has been mentioned in our earlier work. Briefly, we used titanium isopropoxide, acetonitrile, and urea as precursor materials and then subjected them to microwave irradiation. The fabricated nanoparticles were characterized, and further toxicity was analyzed (Ranjan and Ramalingam, 2016; Ranjan et al., 2016a,b).

### Cell Line and Culture Medium

Human colon cancer cells (HCT 116) were obtained from the National Centre for Cell Sciences, Pune, India. HCT cells were subcultured in DMEM medium, supplemented with 10% fetal bovine serum (FBS) and 1% of antibiotic (penicillin-streptomycin). The cells were maintained in a humidified incubator with 5% CO_2_ at 37°C.

### Exposure to TNP

One mg/ml stock suspension of TNPs was diluted in DMEM to concentrations ranging from 1 to 200 μg/ml, respectively. Further subsequent assays, such as cell viability and oxidative stress analysis (glutathione estimation, LDH, etc.) were done with varying concentrations of TNP, and 96- and 6-well cell culture plates and 25-cm^2^ cell culture flasks were used for different assays, having a treatment volume of 0.1, 1, and 2 ml, respectively. The treatment volume was selected to ensure that the concentration of NPs per cm^2^ area was equivalent and uniform in 6- and 96-well plates and 25-cm^2^ flasks for all assays.

### Cell Viability by MTT Assay

Cell viability was observed by MTT (3-(4,5-dimethylthiazol-2-yl)-2,5 diphenyltetrazolium bromide) assay. This assay is based on the ability of the cells to convert soluble MTT dye upon reaction to formazan insoluble crystals. Cells were grown up to 80% confluency and then seeded in 96-well plates at an initial density of 10^4^/well. The cells were treated with varying concentrations of TNPs for 24 h. After the incubation period, media was aspirated from the wells. Cells were incubated with 20 μl of MTT at 5 mg/ml concentration in phosphate buffer saline (PBS) for 4 h. The purple color formazan crystals were dissolved in 100 μl of DMSO and measured in a microtiter plate reader (Readwell Touch, Robonik India Pvt. Ltd., India) at 570 nm (Kuppusamy et al., 2016). The OD value (absorbance) was converted to viability percentage by using the following formula:

% of cell viability = (OD value of treated samples/OD value of control) x 100

Cell proliferation was stated as a cell viability percentage of treated relative to untreated model.

### Neutral Red Uptake (NRU) Assay

For the NRU assay, cells were seeded for 24 h in a 96-well plate at a population of 10^4^ cells per well. The spent media was aspirated and washed with PBS. It was then replaced with fresh media containing test nanoparticles of varying concentrations (0–25 μg/mL). The plates were incubated for different time periods: 6, 12, 24, and 48 h at 37°C in a humidified incubator with a 5% CO_2_ environment. After the incubation period, cells were washed with PBS twice, and serum-free media (100 μL) containing neutral red (100 μg/mL) was added to each well and incubated for 2 h. The cells were washed twice with PBS after the incubation, and 50 μL of dye release agent (a solution of 1% acetic acid, 50% ethanol, and 49% distilled water) was added and incubated for a further 10 min. The optical density was determined at 540 nm on a multiwall spectrophotometer (Readwell Touch, Robonik India Pvt. Ltd., India) (Repetto et al., 2008).

### Morphological Analysis of HCT 116

The morphological changes of control and treated HCT116 cells were observed by staining with crystal violet (Kuppusamy et al., 2016). Briefly, cells were treated with IC_50_ concentration of TNPs for 24 h at 37°C in a 5% CO_2_ atmosphere and observed under an inverted microscope.

### Acridine Orange/Ethidium Bromide Staining

HCT cells were seeded on a coverslip in a 6-well plate. Cells were then treated with IC_50_ concentration of TNP for 24 h. After treatment, media was removed, and cells were washed with PBS. A combined staining of AO (50 mg/mL) and EtBr (5 mg/mL) was then added for 5 min and washed with PBS. It was further examined by a fluorescence microscope (FM 3000, Weswox Optik, Ambala, Haryana, India) at a magnification of 40× (Nair et al., 2011).

### DNA Damage by Hoechst Staining

Hoechst staining was used to observe any morphological changes due to apoptosis. Briefly, HCT116 cells were cultured on cover slips in a 6-well plate and treated with IC_50_ concentration of TNP for 24 h. Further, HCT116 cells were fixed in para-formaldehyde (4% v/v) for 10 min and then incubated with 10 μg/mL of Hoechst 33342 dye for 10 min. Cells were then observed under a fluorescent microscope after the PBS wash (Chu et al., 2012).

### Analysis of Early and Late Apoptosis by Annexin V FITC/PI

HCT 116 cells undergoing early/late apoptosis or necrosis were analyzed by annexin V-FITC and PI staining according to the protocol supplied by the manufacturer (Caymann Chemicals). Briefly, cells were treated with the IC_50_ TNP concentration for 24 h. Cells were harvested, washed with PBS, and resuspended in 100 μL of binding buffer (10 mM HEPES, pH 7.4, 140 mM NaCl, and 2.5 mM CaCl_2_). Cells were then incubated with 50 μL of Annexin V-FITC/propidium iodide staining solution in the dark at room temperature for 10 min. Further, binding buffer (150 μL) was added to it and immediately analyzed in a flow cytometer (BD FACS Calibur Flow Cytometer, Becton, Dickinson and Company, BD Biosciences, Franklin Lakes, NJ, USA). For compensation purposes, cells stained with Annexin V FITC and propidium iodide only were also prepared. Cells were fractioned by virtue of their internal granularity and size based on the signals collected by side and forward scatters, respectively. It can be noted that a two-dimensional dot plot was used to plot the data, and 2.5 × 10^4^ events were acquired within the specific gate for each sample. Four cell subpopulations were identified using a log BB515-A vs. log PE-CF594-A quadrant dot plot: alive, early apoptotic, late apoptotic, and necrotic (Shieh et al., 2010).

### Cell Cycle Analysis by Propidium Iodide

The cell cycle was analyzed by PI staining according to the protocol supplied by the manufacturer (Caymann chemicals). Briefly, treated and untreated cells (containing ~ 10^6^ cells/mL) were harvested, and 70% ethanol was used to fix, adding drop-wise while vortexing. Storage of the fixed sample was done 24 h at 4°C. The ethanol-suspended cells were centrifuged for 5 min at 1,250 rpm at room temperature. After thorough decanting of ethanol, the cell pellet was centrifuged after suspension in 5 mL of cold PBS. Cells were washed two times with PBS to completely remove the ethanol. DNase-free RNase was added to the washed cells after thorough decantation of PBS and incubated at room temperature in darkness for 30 min.

Propidium iodide was then added to the cells and incubated for 15 min. After incubation, PBS (0.5 mL) was used to wash each sample, and the cell cycle was analyzed by using a flow cytometer. After fractionation of cells, 10^4^ events were acquired within a specific gate. Cell distribution in each phase of the cell cycle—G_0_/G_1_, S, and G_2_/M—were determined from the histogram using Flowing Software (Perttu Terho, University of Turku, Finland) (Poniedziałek et al., 2014).

## Oxidative Stress Analysis

### Reactive Oxygen Species (ROS) Generation

DCFH_2_-DA dye was used to observe ROS generation due to TNP treatment. Briefly, cells were cultured onto a coverslip in a 6-well plate. Cells were treated with IC_50_ concentration of TNP for 24 h. Cells were then washed with PBS, and DCFH_2_-DA dye was added to both control and treated cells. Cells were incubated in dye for 30 min and washed with PBS. The ROS generation was then observed under a fluorescent microscope (Han et al., 2014).

### Lipid Peroxidation Estimation

Lipid peroxidation was analyzed according to the protocol supplied by the manufacturer (Caymann Chemicals). Briefly, cells were seeded in 6-well plates and treated with different concentrations of TNPs (0–25 μg/mL) for 24 h. After treatment, cells were harvested, washed two times with PBS, centrifuged at 500 × g with a resuspended pellet in PBS and sonicated further. Lipid hydroperoxides were extracted into chloroform using cell lysate, and ferrous ion–containing solution was then added to the cell extract, which forms ferric ions on reaction with lipid hydroperoxide. After incubation for 10 min, absorbance was measured at 500 nm by UV-Vis spectrophotometer.

### Glutathione Estimation

A tripeptide, glutathione (GSH) serves glutathione transferases as a nucleophilic co-substrate in the detoxification of xenobiotics. In the reduction of hydroperoxides, GSH is an essential electron donor to glutathione peroxidases. An enzymatic recycling method is used for the detection of GSH. In the enzymatic recycling method, 5-thio-2-nitrobenzoic acid (DTNB, Ellman's reagent) reacts with the sulfhydryl group of GSH to produce yellow-colored 5-thio-2-nitrobenzoic acid (TNB). Mixed disulfide of GSH and TNB (GSTNB) is concomitantly produced. Further reduction of GSTNB by glutathione reductase leads to the production of TNB. The concentration of GSH in the sample is directly proportional to the rate of TNB production. Henceforth, for the estimation of GSH in the sample, TNB was measured at 405 nm.

GSH levels in cells were analyzed according to the protocol supplied by the manufacturer (Caymann Chemicals, item no. 703002). Briefly, TNP-treated cells were homogenized in 50 mM, pH 6.0 cold MES buffer containing 1 mM EDTA. The homogenate thus obtained was centrifuged at 10,000 rpm at 4°C for 15 min, and the supernatant was deproteinated; then 50 μl of the samples were transferred to a 96-well plate, and 150 μl of freshly prepared assay cocktail was added. After 25 min of dark incubation of the plates, the absorbance values were measured at 405 nm using a UV-Vis spectrophotometer (AU2701, Systronics Inc., India). A standard curve was prepared using 50 μl of standards having total GSH equivalents ranging from 0 to 16 μM. Total GSH for each sample was then calculated from the standard curve:

(1) $$\begin{array}{r} TotalGSH=2^{*}\frac{\left(Abs-y\;\right)}{m}, \end{array}$$

where Abs = sample absorbance,

Y = *y* intercept,

and m = slope.

Results were expressed as means of at least three replicates ± standard error.

### Mitochondrial Membrane Potential Assay

Enzyme activities of the mitochondrial electron transport chain lead to the generation of potential across the mitochondrial membrane. During the apoptotic process, mitochondrial membrane potential collapses, which coincides with the opening of the pores responsible for the mitochondrial permeability transition. This mitochondrial permeability transition opening leads to the cytochrome c release into the cytosol. In turn, the cytosol-containing cytochrome c triggers the other downstream events in the apoptotic cascade.

JC-10 dye was used to analyze mitochondrial membrane potential. The protocol followed was as per the instructions supplied by the manufacturer (Sigma-Aldrich). Briefly, cells were treated with varying concentrations of TNP for 24 h in a 96-well plate. After treatment, JC-10 dye (50 μl) loading solution was added to each well and incubated for 60 min in the dark. After incubation, 50 μl of assay buffer was added to each well, and fluorescence intensity was measured (λ_ex_ = 490/ λ_em_ = 525 nm) and (λ_ex_ = 540/λ_em_ = 590 nm) for ratio analysis of red and green fluorescence. The ratio of red/green fluorescence was used to estimate MMP.

### Western Blot Analysis

HCT 116 cells were treated with TNPs at different concentrations (0, 30, and 50 μg/mL) for 24 h. After treatment, cells were washed thoroughly using PBS. Cells were then harvested and lysed using lysis buffer (RIPA buffer). It can be noted that the RIPA buffer procured contained a protease inhibitor cocktail (Sigma). The standard Bradford's method was used for the estimation of total cellular proteins, and 50 mg of proteins were separated from control as well as treated groups by using 10% sodium dodecyl sulfate gels and further transferred by electro-blotting to a nitrocellulose membrane. The nitrocellulose membrane was incubated along with primary antibodies specific for Bax, Bcl-2, caspase-3, caspase-9, and β-actin (Abcam, USA). After incubation with a secondary antibody, the protein bands were detected using chemiluminescence (Super Signal West Pico chemiluminescent reagent, Pierce, Rockford, IL) (Lu et al., 2011).

## Results and Discussion

### TNP Synthesis and Characterization

With the recent use of nanoparticles in various fields, it is necessary to evaluate the cytotoxicity of nanoparticles. TNPs are one of the top five nanoparticles synthesized worldwide and produced at the rate of thousands of tons per year (Farner et al., 2019). TNPs, due to their excellent photocatalytic activity, are used for various applications, such as water treatment, bioremediation, medicine, etc. TNPs were fabricated by a novel method—the microwave irradiation–assisted hybrid chemical approach—for improved bioactivity. The nanoparticles were then characterized by different instrumental techniques, and the average particle size was observed to be 28.3 ± 3.1 nm and zeta potential was −35.8 mV. The detailed synthesis protocol and characterization data have already been reported as per earlier reports (Ranjan and Ramalingam, 2016; Ranjan et al., 2016a,b).

### Cytotoxicity Assay

The MTT assay is based on reduction of tetrazolium salts to analyze cell proliferation. The metabolically active cells reduce the yellow color of the MTT in part by dehydrogenase enzymes. NADH and NADPH are generated as reducing equivalents. The intracellular purple formazan thus formed can be quantified by spectrophotometric means. As such, when metabolic events lead to apoptosis or necrosis, the reduction in cell viability can be estimated by this assay (van Meerloo et al., 2011).

**Figure 1:**
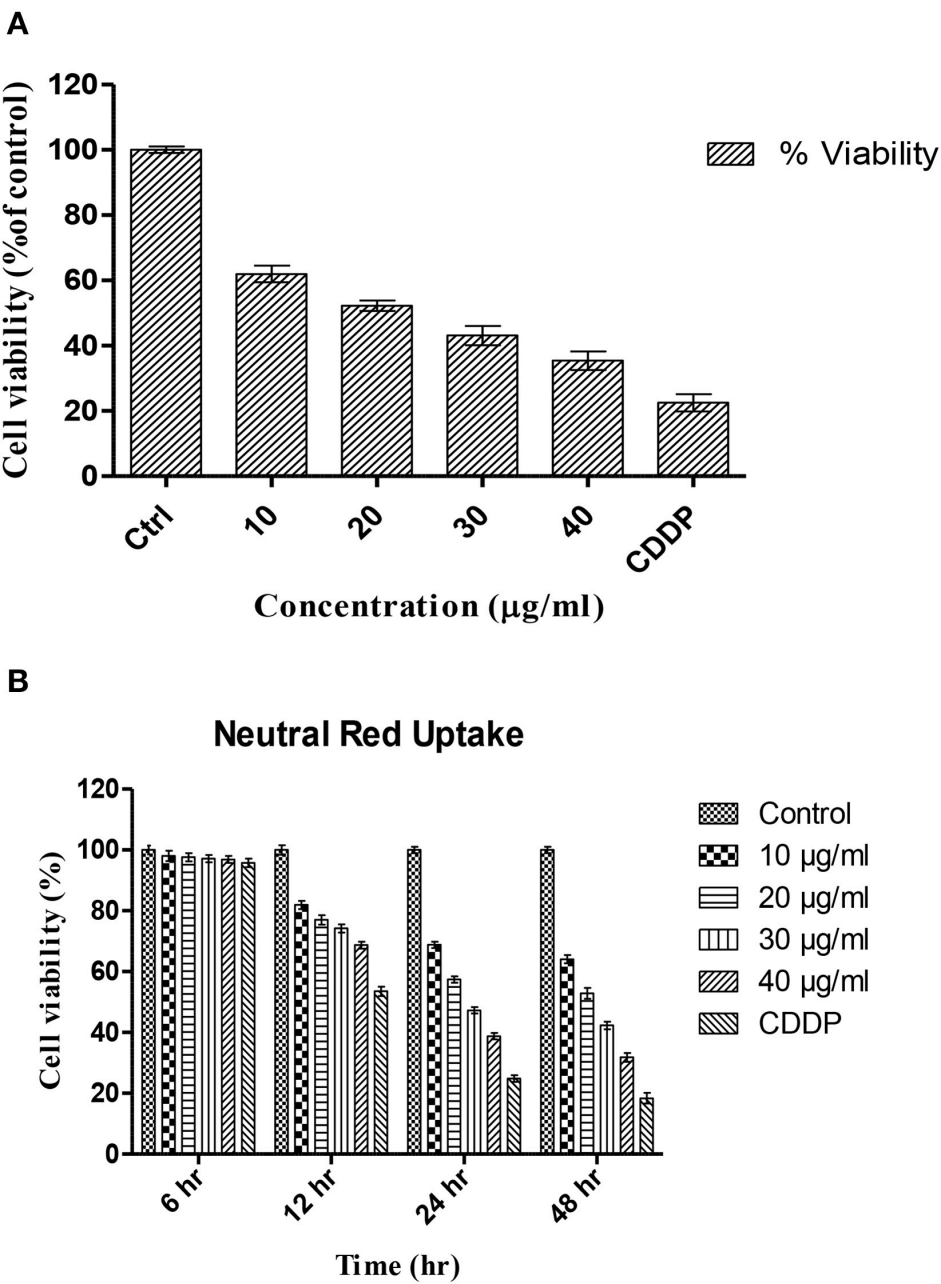
Percentage viability of HCT-116 cells upon treatment with different concentrations of TNPs as analyzed by **(A)** MTT assay and **(B)** Neutral red uptake assay.

After 24 h of incubation, TNPs showed a dose-dependent cytotoxicity on HCT cells. As depicted in Figure 1A, the control was made with 100% viable cells, and the toxicity was calculated accordingly. No significant cytotoxicity was observed after 6 and 12 h exposure. The IC_50_ value of TNP found after a 24 h exposure period was calculated to be 22.97 μg/ml. Our study is in accordance with previously reported works in which a dose-dependent toxicity was observed (Kansara et al., 2015; Bessa et al., 2016; Hanot-Roy et al., 2016). Saquib et al. (2012) report a 24.5% reduction in cell viability by TNP of 30.6 nm at a concentration of 10 μg/ml in human amnion epithelial cells.

However, non-toxic TNPs have also been reported, and they may be due to bigger size or coating of TNP with biocompatible materials. Xie et al. (2016) report that surface modification of TNP with –COOH and –NH_2_ had lesser toxicity than bare TNP. Toniatto et al. (2017) develop an inert material by electro-spinning of polylactic acid with high doses of TNP (1–5 wt%), which showed no mammalian cell toxicity. These observations confirm that, apart from shape and size, surface modification also plays a major role in deciding the cytotoxicity.

### NRU Assay

Neutral red is a weakly cationic dye that penetrates the cell membranes by non-ionic passive diffusion and further concentrates in the lysosomes. In lysosomes, it binds to anionic and/or phosphate groups of the matrix by electrostatic hydrophobic bonds. In dead cells when the pH gradient is reduced, the dye cannot be retained inside the cells. Accordingly, the amount of retained neutral red dye is proportional to the number of viable cells (Repetto et al., 2008).

NRU following 6–48 h exposure to TNP is shown in Figure 1B. The results indicate that the uptake of HCT-116 cells is dependent on dose and also exposure time. With increasing exposure time, a decrease in viability was observed at all concentrations. The percentage of viability was similar to the viability depicted by the MTT assay, indicating that TNP can alter lysosomal integrity. The viability was significantly reduced after 24 h of exposure period. At a concentration of 20 μg/ml, the viability was reduced to 57% against 24% for the positive control.

Dubey et al. (2015) report the IC_50_ value for TNP to be 34.99 ± 0.09 mg/L, which is similar to our IC_50_ value. Both studies indicate a higher IC_50_ value from that obtained from the MTT assay, suggesting that TNP induces more damage on mitochondria than to lysosomes. Vevers et al. also report significant reduction in lysosomal integrity and DNA damage after TNP exposure to a fish cell line (RTG-2 cells) (Vevers and Jha, 2008).

### Morphological Analysis

**Figure 2:**
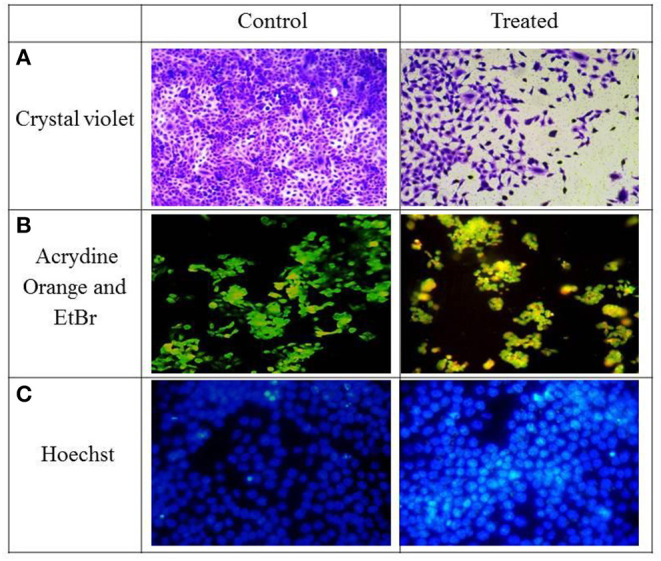
HCT-116 cells—control and treatment with IC_50_ concentration were stained with different dyes **(A)** Crystal violet, **(B)** Acrydine Orange, and EtBr **(C)** Hoechst.

As depicted in Figure 2A, reduction in cell viability was observed after 24 h of treatment. Approximately 50% cell death was observed at a dosage of 22.97 μg/ml, which is the IC_50_ value. Therefore, this dose was selected to explore the mechanisms underlying the morphological changes.

### AO/Etbr Staining

AO can stain both live and dead cells; on the contrary, EtBr can only stain membrane integrity–lost dead cells. Interestingly, the live cells are exited uniformly green, apoptotic cells are also permeable to EtBr and stain orange, and an orange-red stain appears for the necrotic cells. Murugan et al. (2016) also observed apoptotic cells after treatment with 60 μg/mL of concentration in MCF-7 cells. They also observed nuclear shrinkage and blebbing in cells after treatment.

As observed in Figure 2B, green fluorescence was observed in live cells and a yellow to orange color in apoptotic cells. The control cells were mostly green in color although the cells after treatment with IC_50_ concentrations of TNPs had a yellow to orange fluorescence, indicating that cell death occurred via apoptosis. Also, some morphological changes were also observed as compared to the control cells.

### Hoechst Staining

As depicted in Figure 2C, cells stained with Hoechst after TNP treatment induce nuclear material aggregation or condensation, the chromatin margination of which is typical of apoptotic phenomena, whereas a smooth and even-surfaced cellular morphology was observed in the control cells.

Hoechst binds to the minor groove of double-stranded DNA, especially adenine-thymine–rich regions of DNA. On binding with DNA, the fluorescence greatly increases. Because the dye is a membrane permeant, it can stain live cells also. During apoptosis, a cell demonstrates nuclear condensation and DNA fragmentation (Chazotte, 2011). Similar nuclear fragmentation has been observed by TNPs in a human bronchial epithelial cell line of size 75 nm (Chen et al., 2008) and also in a 15-nm size (Hussain et al., 2010)

### Analysis of Early and Late Apoptosis by Annexin-V FITC/PI

The mode of cell death—apoptosis and necrosis—was measured using Annexin-V FITC and propidium iodide. Annexin-V FITC is a fluorescently labeled dye that binds specifically to externalized phosphatidylserine ligands on the cell surface, and this leads to the detection of early apoptotic cells. The apoptotic cells that lose the asymmetry of membranous phospholipids leave phosphatidylserine behind on the plasma membrane outer leaflet. Annexin-V is a calcium-dependent, phospholipid-binding protein having a higher affinity for phosphatidylserine. Therefore, Annexin-V can be used as a sensitive probe to analyze the presence of phosphatidylserine on the phospholipid bilayer and, hence, as an apoptotic marker. On the other hand, propidium iodide is a non-specific DNA (which is excluded by the plasma membrane of living cells) intercalating agent. Thus, it can be used to differentiate between the necrotic cells from apoptotic and living cells by supravital staining without prior permeabilization (Lu et al., 2011).

**Figure 3:**
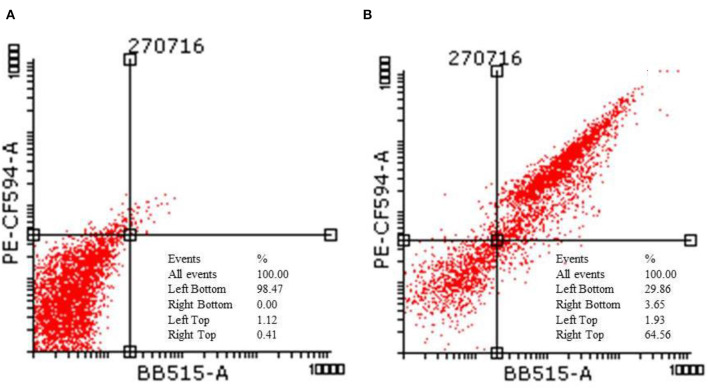
Flow cytometric analysis of HCT-116 **(A)** control and **(B)** treatment cells after staining with annexin V and propidium iodide.

Figures 3A,B corresponds to the representative dot plots of Annexin-V/propidium iodide staining in HCT-116, and also shows the calculated percentages of apoptotic cells. The control had 98% of viable cells. Cells after treating with IC_50_ concentration of TNP showed mostly late apoptosis (64%) and a few cells of early apoptosis (13%).

Liu et al. (2010) demonstrate that the apoptosis pathway in PC12 cells was due to the intracellular ROS. The apoptosis in cells increased from 5.27 to 23.47% after treatment with 50 μg/ml of TNP. However, it decreased to 10.26% after pretreatment with N-(mercaptopropionyl)-glycine (a ROS scavenger). This indicates that ROS can trigger apoptosis. Apart from the synthesis method, particle type also determines if the cells will undergo apoptosis or necrosis. Wu et al. (2010) demonstrate that, for anatase TNP, the cell death mechanism could be apoptosis or necrosis, and rutile TNPs followed the apoptosis pathway only.

### Cell Cycle Analysis by Propidium Iodide

**Figure 4:**
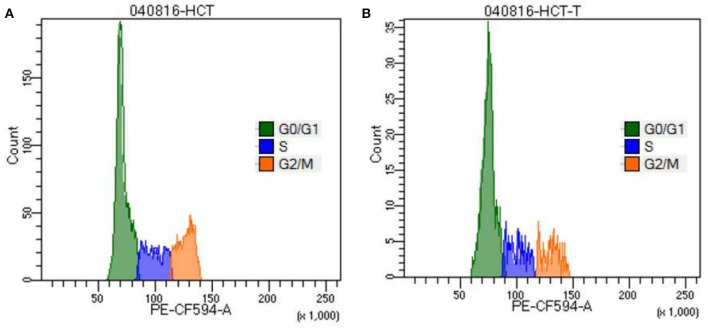
Cell cycle analysis of HCT-116 **(A)** control and **(B)** treatment cells by staining with propidium iodide.

PI is an intercalating dye that binds to the DNA. Thus, it was used to study the DNA content in cell cycle phases. As depicted in Figures 4A,B, TNPs cause a significant increase in the proportion of cells that are arrested in the G_0_/G_1_ phase (61%) as compared to control (55%), whereas there is a decrease in the number of cells entering the S phase (17%) and G_2_/M phase (16%) as compared with control (19 and 23%) respectively. Due to the DNA damage, cells undergo programmed cell death, which leads to an elevation in cell accumulation in the G_1_ phase.

TNPs were observed to have G_1_ cell cycle arrest in cell lines. Our results are in agreement with the earlier cited literature that the cell cycle is arrested at the G_1_ phase after treatment with TNP (Kocbek et al., 2010; Chen et al., 2016). However, some reports also observed the cell cycle arrest in the G_2_/M phase (Wu et al., 2010; Kansara et al., 2015). This may be due to the fact that the toxicity of the nanoparticle depends not only on size and shape but also the synthesis method followed. This also concerns that the current toxicological protocols have to be modified so that the exact toxicity mechanism can be predicted for different nanoparticles.

### ROS Assay

**Figure 5:**
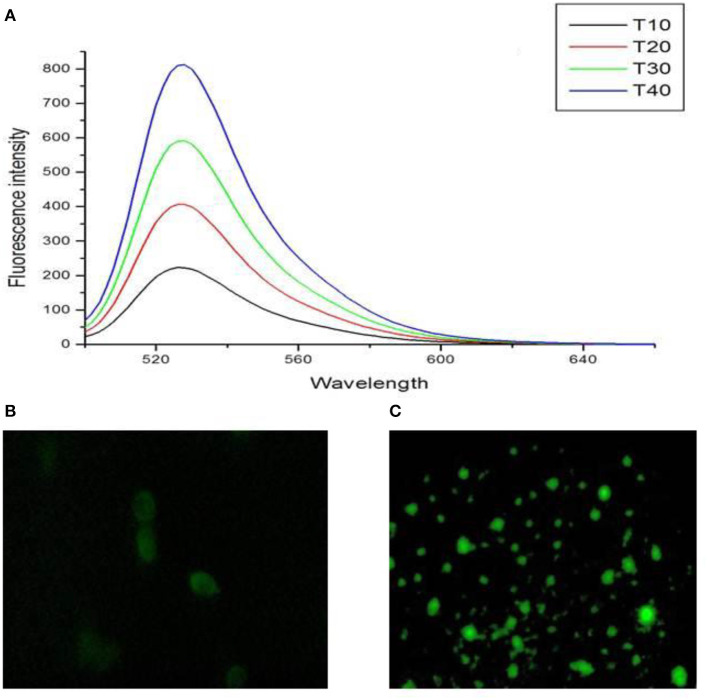
**(A)** Dose dependent increase in intracellular ROS levels and fluorescent microscopic image of HCT-116 **(B)** control and **(C)** treatment cells after staining with DCFH_2_-DA dye.

The cells exposed to IC_50_ (22.97 μg/ml) of TNP for 24 h indicated a significant increase in the generation of ROS in a dose-dependent manner (Figure 5A), and fluorescent microscopy data revealed induction of intracellular ROS generation (Figures 5B,C).

Recent cancer research suggests ROS generation through oxidative stress as a most common mechanistic pathway of most of the apoptotic stimuli. For the initiation and execution of apoptosis, ROS has been an essential signaling molecule (Shafagh et al., 2015; Fu et al., 2017). In a study by Maurer-Jones et al. (2012), an increase in ROS production in mouse peritoneal mast cells after exposure to TNPs of 11 nm was observed. They also report that nanoparticle-induced ROS production is directly related to exocytosis perturbations.

### Lipid Peroxidation Estimation

Lipid peroxidation is a naturally generated process in the body in small amounts, mainly by the several ROS effects (hydroxyl radical, hydrogen peroxide, etc.). However, the excess ROS production results in unwanted oxidation of the lipid bilayer of the cell, leading to lipid peroxidation and ultimately resulting in membrane damage and rupture. The hydroperoxides MDA (malondialdehyde) and 4-hydroxy non-enal are produced during the peroxidation of unsaturated fatty acid by oxygen-based free radicals. This lipid peroxidation intensity is related to the concentration of ROS (Dutta et al., 2012).

**Figure 6:**
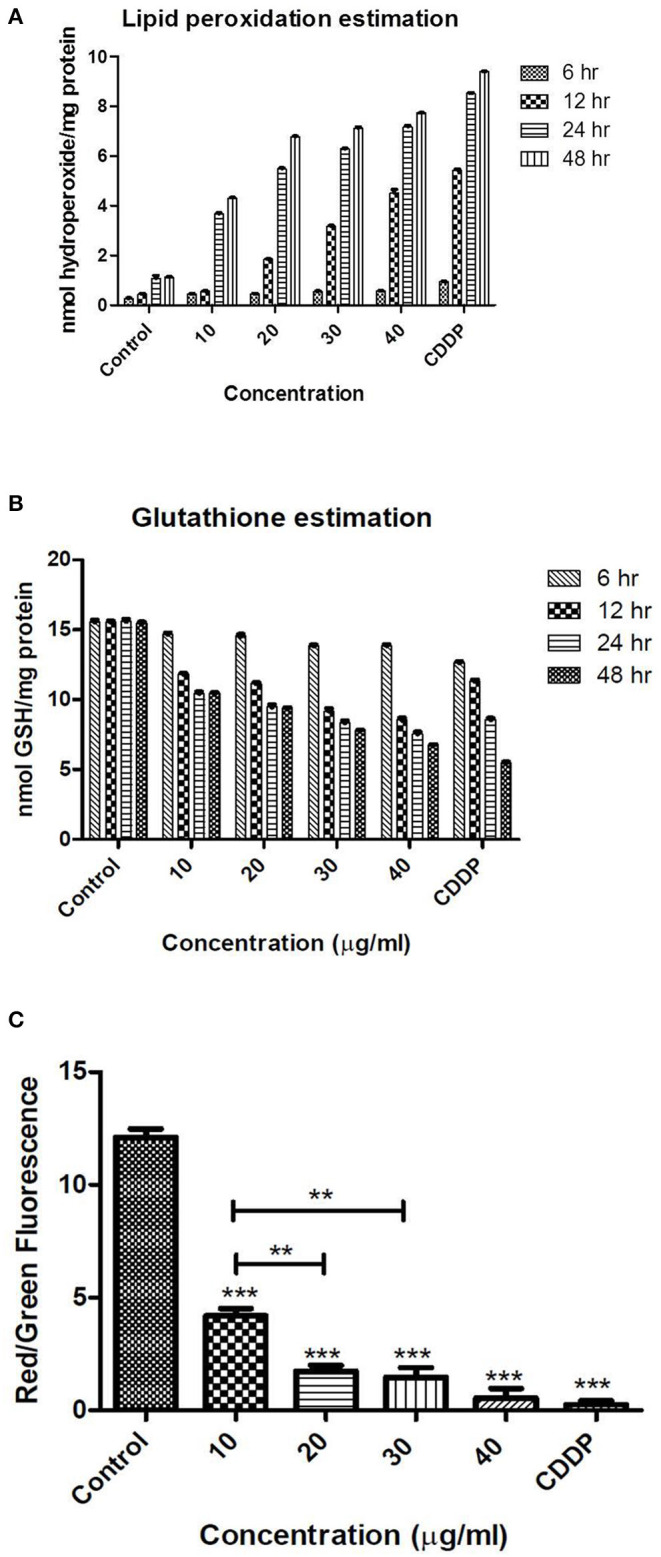
Levels of oxidative stress as analyzed by **(A)** lipid peroxidation **(B)** GSH levels **(C)** mitochondrial membrane potential (staining with JC-1 dye) after treatment with TNPs at different exposure time and concentrations. **, *** indicates the significance level.

An estimation of hydroperoxides is a direct measurement of lipid peroxidation in the cell as shown in Figure 6A. The control had a negligible amount of hydroperoxide; however, a significant increase was observed after 24 h of treatment. In the positive control, a significant increase was observed after 12 h of treatment also. Lipid peroxidation was observed in other reports also after treatment with TNPs in cell lines and in rat models also. Rizk et al. (2017) report significant increased levels of MDA in rat hepatic tissue after treatment with TNP of 14 days. They also observed significantly increased levels of ALT (alanine transaminase), AST (aspartate aminotransferase), catalase, and nitric oxide (NO). Wang et al. (2013b) also demonstrate that TNPs induced excessive ROS and MDA production on rat synovial cell line 364. They also observed nuclear shrinkage and mitochondrial swelling of the cells with a dose of 300 μg/ml.

### Glutathoine Estimation

Glutathione (GSH) is one of the important cellular antioxidants that is made up of three amino acids, namely glycine, glutamic acid, and cysteine, Every body cell has the ability for glutathione synthesis, but it is present in higher concentrations in some specific organs, such as the intestinal tract, lungs, and liver. Glutathione major functions include detoxification, antioxidation, signal transduction, and strengthening the immune system under stressful conditions. The major reason behind the diverse function of GSH is presence of a sulfhydryl group containing amino acid cysteine, which enables the GSH to chelate and detoxify harmful substances. Its ability to donate electrons makes it an important and useful antioxidative agent, and in that process, GSH oxidizes to glutathione disulfide (GSSG) form. GSH can prevent oxidative cellular damage from free radicals by donating an electron to a free radical and, thus, quenching the unstable and highly reactive free radical. GSSG is reduced to GSH by NADPH, an electron donor in this reaction, a reaction catalyzed by glutathione reductase. Thus, the ratio of reduced and oxidized glutathione acts as an indicator of cytotoxicologically mediated oxidative damage (Vagula and Konieczko, 2012).

Altered GSH levels are an indication of functional damage of cells. Irrespective of the time period, the control exhibited the same amount of GSH. However, unlike the ROS and MDA levels, where a significant increase was observed after 24 h, the level of GSH was decreased significantly after 12 h as depicted in Figure 6B. At the highest concentration of 40 μg/ml, a significant decrease in GSH was observed after 12 h (8.62 ± 0.054 nmol GSH/mg protein), which is less than the levels of GSH obtained after treatment with positive control (11.25 ± 0.082 nmol GSH/mg protein). This indicates that, at higher doses, oxidative stress is generated even after 12 h of treatment. Our results are in agreement with the earlier reports in which decreased levels of GSH are reported after treatment with TNPs.

Wang et al. (2013a) report a dose-dependent reduction in levels of total GSH and reduced GSH contents in a rat synovial cell line (RSC-364). They also observe a decrease in levels of oxidized glutathione, which indicates an interruption of the cellular redox system. Dubey et al. (2015) report that total GSH was depleted to 176.36, 134.99, and 114.48% of control in WAG cells for TNPs treated in doses of 25.29 ± 0.12, 34.99 ± 0.09, and 35.06 ± 0.09 mg/l, respectively.

### Mitochondrial Membrane Potential

Deviations in the permeability of the mitochondrial membrane are a hallmark for apoptotic processes. Cellular death is importantly regulated by mitochondria, and a permeability change of the mitochondrial membrane is known to be an early event in apoptosis. The mitochondrion releases many pro-apoptotic proteins into the cytoplasm, and further formation of a permeability transition pore takes place in the mitochondrial membrane. To elucidate mitochondrial involvement in the TNP-induced apoptotic process, permeability alterations of the mitochondrial membrane were evaluated using a staining kit for mitochondria. The dye JC-1 (5,5′,6,6′-tetrachloro-1,1′,3,3′-tetraethyl benzimidazolocarbocyanine iodide) is a convenient and faster method for detecting any changes in membrane permeability of living cell mitochondria. In normal cells, the dye JC-1 concentrates in the mitochondrial matrix due to the electrochemical potential gradient of JC1. In the mitochondrial matrix, it forms J-aggregates, red fluorescent aggregates. Dissipation of the mitochondrial membrane potential prevents JC-1 dye accumulation in the mitochondria, and thus, the JC-1 dye is dispersed throughout the entire cell. This leads to a shift from red (J-aggregates) to green fluorescence (JC-1 monomers), which ultimately indicates the depolarization of the mitochondrial membrane (Zhao et al., 2009).

The cellular apoptotic process usually occurs when there is an internal environmental destruction of cells. Reactive oxygen species generation is an important factor in the apoptosis of cells. Mitochondrial membrane permeability is induced by excessive ROS generation and further damages the respiratory chain to trigger cellular apoptosis. We demonstrate our results as a red to green fluorescence ratio, where a higher ratio indicates more viability and a lower ratio indicates apoptosis. The control had a high red to green fluorescence ratio. The cells after treatment with IC_50_ concentration of TNP showed a dose-dependent decrease in the red to green fluorescence ratio, indicating significant effect on the mitochondrial membrane permeability as shown in Figure 6C.

Wang et al. (2015) determine mitochondrial membrane permeability in A549 cells by rhodamine 123 dye and observe a significant dose-dependent increase in green fluorescence. They observe mitochondrial membrane depolarization and apoptosis via activation of the intrinsic mitochondrial pathway in A549 cells. Zhao et al. (2009) also demonstrate mitochondrial membrane depolarization in a mouse epidermal cell line (JB6) after treatment with TNPs of size 21 nm. They also observe release of cytochrome c from mitochondria to cytosol as detected by Western blot analysis, and TNPs induce apoptosis by activation of caspase-8/Bid pathway.

### Western Blot Analysis

**Figure 7:**
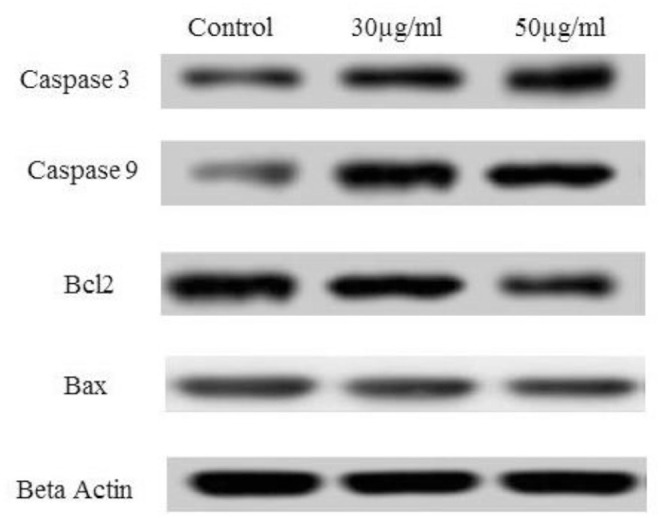
Effect of TNP on different anti-apoptotic and pro-apoptotic proteins such as caspase3, caspase 9, Bcl2, Bax and control as beta actin.

As depicted in our study, TNP induces ROS, which triggers cell-cycle arrest. This enables the cells to undergo either a self-mediated apoptotic process or DNA repair. Alteration of certain proteins triggers the mitochondrial pathway–mediated cellular apoptosis. Members of the Bcl-2 protein family are associated with the regulation of mitochondrial membrane integrity. Hence, the relative ratio alteration of Bax/Bcl-2 could be the indicator for determining the level of cellular stress required for inducing the apoptotic process (Shafagh et al., 2015). Bcl-2 proteins are a family of proteins with pro- and anti-apoptotic proteins involved in apoptosis. In our study, we observe that no effect was observed on Bax (pro-apoptotic protein) after TNP treatment although Bcl-2 (anti-apoptotic protein) was downregulated as depicted in Figure 7. It can be noted that the Bax/Bcl ratio is important as it determines the susceptibility to apoptosis. Thus, Bcl can alter the ratio of Bax/Bcl. Certain studies report that the downregulation of Bcl alone is sufficient for triggering apoptosis (Shafagh et al., 2015).

Caspases are known to play a vital role in both execution and initiation of apoptosis and are activated during apoptosis in many cells. As per earlier reports, activated caspase-3 is important and vital for DNA damage and apoptosis of cells. Alteration in the Bax/Bcl ratio induces outer mitochondrial membrane permeabilization, which releases soluble proteins into the cytosol from the intermembrane space, where they promote activation of caspase. Caspase-9 activates caspase-3 (effector caspase), which cleaves substrates at aspartate residues, and activation of this proteolytic activity appears to be an event in apoptosis (Youle and Strasser, 2008; Alarifi et al., 2017). In our study, caspase-3 and caspase-9 were activated after treatment with IC_50_ concentration of TNP.

A similar observation is reported in earlier cited literature (Xia et al., 2008; Zhao et al., 2009; Yoo et al., 2012; Zhu et al., 2012) in which caspase-3 and−9 were activated, and a downregulation of Bcl-2 was observed. However, few reports state that Bax is activated by TNP treatment (Yoo et al., 2012), and in some reports, apoptosis is observed independently of Bax activation (Zhu et al., 2012). This may be due to the fact that apoptosis is mediated by alteration in the Bax/Bcl ratio and not either of them alone. Thus, treatment with TNP mediates apoptosis by the Bax/Bcl pathway. This alteration leads to formation of a permeability transition pore in mitochondria and ultimately release of cytochrome c, which, in turn, activates the caspase cascade.

## Conclusion

TNP is greatly used in different sectors that impact society, such as the food, agriculture, environment, medicine, and biomedical sectors. Apart from its applications, the toxic effects of TNPs are recently identified for safe use of TNPs. The toxicity of nanomaterials greatly depends on the fabrication methods, which ultimately alter the size, shape, crystal structures, and charge distributions. In our present study, we analyze the detailed cytotoxicological effects and mechanisms of action of titanium dioxide nanoparticles synthesized from a microwave irradiation-assisted hybrid chemical approach. The cytotoxic impact is analyzed on human colon cancer cells, HCT116. Our results indicate that, after exposure to TNPs, oxidative stress and ROS generation is induced in cells that leads to apoptosis. DNA damage and cell-cycle arrest in the G_0_ phase is also observed. Thus, this work provides a deep insight into the mechanism of action of TNP-induced apoptosis in colon cancer cell lines and can be further used to design the safety of TNPs for various other consumer products. Also, detailed toxicological studies need to be done for other nanomaterials of different shape, size, and crystalinity for their safe applications.

## Data Availability Statement

The datasets generated for this study are available on request to the corresponding author.

## Author Contributions

SR and ND have done the experimental work and analyzed the data. DM and CR have designed the concept and helped in manuscript writing.

## Conflict of Interest

The authors declare that the research was conducted in the absence of any commercial or financial relationships that could be construed as a potential conflict of interest.
